# Seroprevalence of Arboviruses and Genetic Characterization of Orbiviruses in Sloths from Western Panama

**DOI:** 10.3390/v17111507

**Published:** 2025-11-17

**Authors:** Rita Corrales, Yamilka Díaz, Vanessa Pineda, Yaneth Pitti, Lisseth Saenz, Jean-Paul Carrera, Celestino Aguilar, Alexander Martínez, Maria Chen-Germán, Kathryn A. Hanley, Nikos Vasilakis, Robert B. Tesh, Azael Saldaña, Sandra López-Vergès

**Affiliations:** 1 Department of Research in Virology and Biotechnology, Gorgas Memorial Institute for Health Studies, Panama City 0816, Panama; rcorrales@gorgas.gob.pa (R.C.); ydiaz@gorgas.gob.pa (Y.D.); ypitti@gorgas.gob.pa (Y.P.); lsaenz@gorgas.gob.pa (L.S.); jcarrera@crivb.org.pa (J.-P.C.); mchen@gorgas.gob.pa (M.C.-G.); 2 Master Program in Biomedical Sciences, Faculty of Medicine, University of Panama, Panama City 0801, Panama; aguilar.smng@gmail.com (C.A.); azasal@hotmail.com (A.S.); 3 Department of Research in Parasitology, Gorgas Memorial Institute for Health Studies, Panama City 0816, Panama; vpineda@gorgas.gob.pa; 4 Centro Carson de Investigación en Salud y Ecosistemas (Carson Institute), Darien 12026, Panama; 5 Centro Regional de Innovación en Vacunas y Biofármacos (CRIVB AIP), Ciudad del Saber, Panama City 0826, Panama; 6 Department of Human Microbiology and Immunology, Facultad de Medicina, Universidad de Panamá, Panama City 0819-07289, Panama; almartinez@gorgas.gob.pa; 7 Department of Genomics and Proteomics, Gorgas Memorial Institute for Health Studies, Panama City 0816, Panama; 8 Sistema Nacional de Investigación SNI-AIP, Ciudad del Saber, Panama City 0826, Panama; 9 Department of Biology, New Mexico State University, Las Cruces, NM 88003, USA; khanley@nmsu.edu; 10 Department of Pathology, University of Texas Medical Branch, Galveston, TX 77555, USA; nivasila@utmb.edu (N.V.); rbtesh22@gmail.com (R.B.T.); 11 Center for Vector-Borne and Zoonotic Diseases, University of Texas Medical Branch, Galveston, TX 77555, USA; 12 Institute for Human Infection and Immunity, University of Texas Medical Branch, Galveston, TX 77555, USA; 13 Center for Research and Diagnosis of Parasitic Diseases (CIDEP), Faculty of Medicine, University of Panama, Panama City 0801, Panama

**Keywords:** sloth, arboviruses, seroprevalence, orbivirus, Changuinola virus, spillover risk

## Abstract

Arthropod-borne viruses (arboviruses) are an increasingly significant threat to public health in tropical regions. In this study, we investigated the seroprevalence of various arboviruses in two species of sloth (*Choloepus hoffmanni* and *Bradypus variegatus*) in rural and peri-urban areas of Western Panama province. Between 2013 and 2018, blood samples from 60 sloths were tested for neutralizing antibodies against ten arboviruses. Significant variation in seroprevalence of different arboviruses was observed: 6.7% of sloths were seropositive for Madariaga virus, 6.7% for Venezuelan equine encephalitis virus, and 4.8% for Oropouche virus, while none were seropositive for dengue type 2, Zika, chikungunya, Una, Mayaro, or Punta Toro viruses. Notably, two Changuinola virus (CGLV) strains, which were previously isolated from Panamanian sloths in the 1970s, showed high seroprevalence: Pansloth 149 (23.3%) and D50 (53.3%). Given the high seroprevalence detected in our study and the lack of genomic characterization of the historical Pansloth 149 isolate, we performed next-generation sequencing of its complete genome using Illumina technology to understand its genetic diversity and evolutionary relationship with other CGLV strains.

## 1. Introduction

Arthropod-borne viruses (arboviruses), including dengue (DENV), Zika (ZIKV), and chikungunya viruses (CHIKV), are a growing public health concern in tropical regions of Latin America [1]. Of the more than 500 known arboviruses, approximately 150 are pathogenic to humans [2]. Most of these are zoonotic, which the World Health Organization (WHO) defines as diseases that are naturally transmitted between non-human animals and humans, either directly or via arthropod vectors [3]. These cross-species transmissions present a significant public health challenge as emerging zoonotic infections are difficult to detect and control [4]. This surveillance gap poses significant risks to both human and veterinary health, particularly since some arboviruses (e.g., DENV, CHIKV, and ZIKV) have demonstrated the potential to transition to human-endemic transmission cycles [5,6], while even purely zoonotic arboviruses (e.g., West Nile virus (WNV), Oropouche virus (OROV)) can infect large numbers of people following extreme climate events or translocation [5,7,8].

Comprehensive surveillance of wild animal populations is essential for characterizing the transmission cycles of arboviruses in sylvatic environments. The One Health approach, which integrates the human, animal, and environmental health sectors, is vital for developing effective prevention and control strategies for emerging arboviral diseases [9]. Wild reservoirs are necessary for the persistence of zoonoses, and the intensity of transmission within the reservoir and spillover to humans depends not only on vector densities but also on the density of animal reservoirs [10].

Since the 1970s, studies in Panama have demonstrated the relationship between arboviruses and their vertebrate and arthropod hosts, as well as their pathogenesis and associated ecological interactions [11]. Various arboviruses capable of causing outbreaks have been identified in Panama, including DENV, Venezuelan equine encephalitis virus (VEEV), Madariaga virus (MADV, formerly known as Eastern equine encephalitis virus from South America (Latin America), EEEV-SA), CHIKV, ZIKV, OROV, and the Punta Toro virus group (PTV) [12,13,14]. Various arthropod vectors of these and other arboviruses, such as mosquitoes, sandflies, and ticks, frequently feed on sloths [15,16]. Moreover, several isolates of Changuinola-like virus (CGLV) were obtained from sloths in Panama in the 1960s–1970s, suggesting that these animals may serve as natural reservoirs or at least interact frequently with these viruses [17,18].

The CGLV serogroup comprises a diverse range of orbiviruses (genus *Orbivirus*, family *Sedoreoviridae*) [19]. These double-stranded RNA viruses have segmented genomes consisting of ten segments that encode structural and non-structural proteins. They are primarily transmitted by hematophagous arthropods such as midges, mosquitoes, and sandflies. They have been isolated from various vertebrate hosts and arthropod vectors in Central and South America [17,18,19]. The segmented genome structure of orbiviruses facilitates genetic reassortment when multiple viral strains co-infect the same host, potentially leading to the emergence of variants with altered host range, virulence, or transmission efficiency. Despite their discovery decades ago, the genetic characteristics, evolutionary dynamics, and public health significance of Panamanian CGLV strains remain poorly understood.

Our study aimed first to evaluate the seroprevalence of arboviruses that circulate in Panama and cause human disease [12,13,14], as well as the two CGLV strains (Pansloth149 and D50) previously isolated from free-living *Choloepus hoffmanni* and *Bradypus variegatus* sloths in western Panama province, a region with a fast urbanization rate [20,21]. Given that the PanSloth 149 CGLV strain’s complete genome has not been characterized, the study also conducted next-generation sequencing and phylogenetic analysis of its genome to better understand its potential as a zoonotic pathogen.

This study advances our understanding of the eco-epidemiology of these pathogens and provides baseline data for evaluating their potential public health significance.

## 2. Materials and Methods

### 2.1. Ethical Considerations

The study was authorized by the Ministry of the Environment of Panama (MiAmbiente) (permit code SE/A-101-13, approved on 3 January 2013) and the property owners from the three communities. It was also approved by the Institutional Committee for the Ethical Use and Care of Animals at the Gorgas Memorial Institute for Health Studies (GMI) (permit number CIUCAL 01/2021, approved on 19 July 2021). The study has been registered in the RESEGIS platform of DIGESA from the Ministry of Health in Panama under number 3834.

### 2.2. Sample Collection

**Figure 1:**
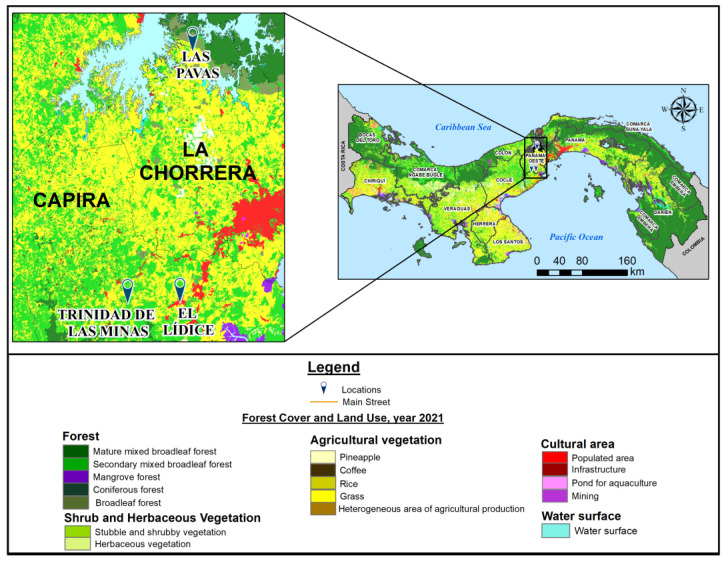
Panama land cover and forest distribution (2021). The color coding illustrates different types of forest (mature, secondary, mangrove, coniferous, and broadleaf), and of vegetation used for agriculture, such as coffee, rice, and pineapple. Zones dedicated to infrastructure, mining, agriculture, population area, and surface water are also shown. Red zones represent urban areas. The enlargement map focuses on the study area, and the sites where sloths were captured are indicated with blue pins. This map was created in ArcGIS Desktop (v. 10.7.1, Esri, Redlands, CA, USA) using shapefiles of forest cover, land use from the Ministry of Environment (MiAmbiente, https://sinia.gob.pa/suelos, accessed on 6 December 2022), and administrative boundaries from the National Institute of Statistics and Census of Panama (INEC, https://www.inec.gob.pa, accessed on 6 December 2022), with marker adjustments for improved visualization.

Between 2013 and 2018, a total of 60 blood samples were collected from two species of sloth, *C. hoffmanni* (*n* = 55) and *B. variegatus* (*n* = 5). The study was conducted in three rural communities in Western Panama province (Panama Oeste), a province that experienced rapid urbanization, with the urban area increasing by around 88% between 2000 and 2015 (https://dpu.mupa.gob.pa/wp-content/uploads/2017/06/Informe_Final_CE3_14012016.pdf, accessed on 4 September 2025). No recaptures occurred during the sampling period. Two of the communities, Lidice and Trinidad de las Minas, are located in the district of Capira (Figure 1). The third community, Las Pavas, is located in the district of La Chorrera. Table 1 provides a detailed description of each community, including geographic coordinates, population density, altitude, and distance from Panama City.

### 2.3. Sloth Trapping and Sample Collection

Wild sloths were caught manually by trained technical assistants who climbed up to reach the animals. The sloths were placed in burlap sacks and transported to a temporary field station, which was established within the community at a distance of no more than 1 km from the capture site, for blood sampling. The field station was equipped with necessary biosafety measures, personal protective equipment, and disinfectant. All procedures were conducted by a veterinary and in accordance with biosafety level (BSL-2) practices to minimize the risk of exposure to potentially zoonotic pathogens. Only adult specimens were included in the study, and all captured individuals were systematically marked with numbered tags to enable individual identification and prevent the inclusion of recaptured animals. Blood samples were taken from the sloths while they were anesthetized with 2.5 mg/kg of ketamine hydrochloride as described by Hanley et al. [22]. A small area of the forearm was shaved to locate the cephalic and ulnar collateral veins. Then, 1 mL of blood was drawn into serum collection tubes using a 23Gx 1” needle. The tubes were then centrifuged at 3500 rpm for 10 min at room temperature. After the centrifugation at the field station, the serum samples were immediately placed in cryovials and stored in a dry ice container for transportation. Samples were preserved at a −80 °C freezer in the Department of Research in Parasitology at the Gorgas Memorial Institute (GMI) until further processing. The serum was used for serological and molecular testing, as well as for viral isolation attempts, as described in Section 2.4 and Section 2.5. Once the samples had been taken and the sloth was awake, it was released back to its original location.

### 2.4. Serological Testing

The viral isolates used in the experiments were produced and titrated in Vero cells (ATCC CCL-81), which were cultured in minimum essential medium (MEM 1X) (Gibco, Grand Island, NY, USA, Cat. No. 11095-080), supplemented with 10% fetal bovine serum (FBS) (Gibco, Grand Island, NY, USA, Cat 16000-044), 1% penicillin/streptomycin (P/S) (Gibco, Grand Island, NY, USA, Cat. No. 12140-122) and 0.5% amphotericin B (Gibco, Grand Island, NY, USA, Cat. 15290-018), as previously described [14]. The viral strains used in this study included representatives from five virus genera (Table 2). The viruses were either isolated at the GMI or obtained from the World Reference Center for Emerging Viruses and Arboviruses (WRCEVA) at the University of Texas Medical Branch (UTMB).

Neutralizing antibodies against these viruses were tested using the plaque reduction neutralization test (PRNT), as previously described [14], in the BSL-2+ laboratory for most arboviruses. MADV was handled in the BSL-3 laboratory at GMI. Heat-inactivated serum samples were serially diluted in a 1:2 ratio, mixed with viral isolates at 800 PFU/mL in a 1:1 ratio, and incubated at 4 °C overnight. The sera/virus mixture was then used to infect Vero cells, which were cultured in MEM supplemented with 10% FBS, 1% P/S, and 0.5% amphotericin B. The cells were allowed to reach 80–90% confluence before infection. The infected cells were incubated at 37 °C and 5% CO_2_ for 1 h, before adding 1 mL of MEM supplemented with 2% FBS, 1% P/S, and 0.5% amphotericin B, and mixed with 0.6% agarose (Promega Corporation, WI, USA, Cat. No. V3125) for alfaviruses, phleboviruses, orthobunyaviruses, and orbiviruses, or with 1.6% carboxymethylcellulose (Sigma-Aldrich, St. Louis, MO, USA, Cat. No. C5678) for flaviviruses. The cells were then incubated at 37 °C and 5% CO_2_ until plaques were detected in the control wells infected with the virus without sera. Once plaques were observed, the cells were fixed with 2% formaldehyde, stained with crystal violet 0.5% (Sigma-Aldrich, St. Louis, MO, USA, Cat. No. V5265) in ethanol, and washed with water. The highest dilution reducing plaque counts by >80% (PRNT_80_) compared to the control virus without sera was considered the neutralization titer. Samples were considered positive at a PRNT_80_ titer of ≥1:20. PRNT was conducted on 60 sloth serum samples for all analyzed arboviruses; however, only 42 samples were analyzed for OROV due to limitations in sample volume. Seroprevalence was calculated as the proportion of positive samples, with a 95% confidence interval (CI) estimated using the binomial distribution method to account for sampling uncertainty. Bivariate associations between sloth characteristics and arbovirus seroprevalence were evaluated using a contingency table analysis.

### 2.5. Molecular Testing and Viral Isolation from Sera Samples

Viral RNA was extracted from sloth serum samples using a QIAamp Viral RNA Mini Kit (QIAGEN, Hilden, Germany, Cat. No. 52906) [31]. Detection of flaviviruses, phleboviruses, and alphaviruses was performed using genus-specific RT-PCRs following the protocols implemented for human and non-human passive surveillance of arboviruses [12,32,33,34]. Detection of *Orbivirus* and *Orthobunyavirus* was based on viral isolation, as genus-specific primers with the required sensitivity and specificity were unavailable at the time of sample processing.

Viral isolation from sloth sera was performed using Vero cells cultured in MEM supplemented with 10% FBS, 1% P/S, and 0.5% amphotericin B. Briefly, confluent Vero cell monolayers were inoculated with 200 µL of undiluted serum and incubated at 37 °C in 5% CO_2_. The cells were observed for 10 days to check for the development of a cytopathic effect (CPE), as previously described [14]. No CPE was observed in any of the inoculated cultures.

Metagenomic sequencing was planned for CPE-positive samples to identify potential novel or unexpected viruses, including orbiviruses and orthobunyaviruses. However, given the absence of viral isolation or RT-PCR detection, metagenomic analysis was not performed on these samples.

### 2.6. Genetic Characterization of an Orbivirus from Sloths

Viral RNA was extracted from the culture supernatant of infected Vero cells. The cells were cultured, and when the cell monolayer reached 90% confluency, they were infected with the Pansloth149 (p149) or D50 viruses at a multiplicity of infection (MOI) of 2 in MEM supplemented with 2% FBS, 1% P/S, and 0.5% amphotericin B. The cells were then incubated at 37 °C in 5% CO_2_ for 4 days until 3+ CPE (corresponding to >75% of cells exhibiting CPE) was observed.

Viral cultures were harvested by mechanically scraping the monolayer, then centrifuging at 2000 rpm for 10 min in 15 mL tubes to maximize viral recovery. The supernatant was then concentrated using 100 kDa Amicon Ultra centrifugal filter units (Millipore, Burlington, MA, USA, Cat. UFC910009) to obtain higher viral titers. Viral RNA was extracted using a MagMAX™ Viral/Pathogen II Nucleic Acid Isolation Kit (Thermo Fisher Scientific, Waltham, MA, USA, Cat. No. A48383) in a KingFisher Flex (Thermo Fisher Scientific, Waltham, MA, USA, Cat. No. 5400610). Contaminating DNA was removed using DNase I (Invitrogen, Carlsbad, CA, USA, Cat. No. AM2238). Then, the RNA was concentrated, and the salts that could inhibit the subsequent steps were removed using a precipitation protocol involving sodium acetate and ethanol [35]. Complementary DNA (cDNA) was produced using the Superscript IV First-Strand Synthesis System protocol (Invitrogen, Carlsbad, CA, USA, Cat. No. 15327696) and the Non-Directional RNA Second Strand Synthesis Module protocol (New England Biolabs, Ipswich, MA, USA, Cat. No. E6111S). Samples were purified using an Ampure XP beads-based protocol (Beckman Coulter, Brea, CA, USA, Cat. No. A63881). Genome amplification using an Illustra GenomiPhi V2 DNA Amplification kit (Cytiva, Marlborough, MA, USA, Cat. No. 25660030).

Library preparation was performed using the Nextera XT library prep kit (Illumina, San Diego, CA, USA) according to the manufacturer’s instructions. Runs were conducted at the Genomics and Proteomics Department of GMI, on the MiSeq platform (Illumina, San Diego, CA, USA) using 500 cycles to produce 2 × 250 bp paired-end reads. The removal of adapter sequences from the reads was performed using FASTP v. 0.23.2 software [36], and de novo genome assembly was carried out using SPAdes v3.15.4 with default parameters [37]. The contigs were then put into NCBI website Nucleotide BLAST (https://blast.ncbi.nlm.nih.gov/Blast.cgi?PROGRAM=blastn&PAGE_TYPE=BlastSearch&LINK_LOC=blasthome, accessed on 9 March 2024) to check for Changuinola virus (CGLV) hits. Ten of the 349 contigs were positive and aligned accordingly for each segment, accordingly, using MAFFT (v.7.490 34, Osaka University, Japan). Furthermore, the contigs representing the CGLV genome segments were validated by mapping the quality-filtered reads using Bowtie2 and visually inspecting the alignments in Geneious Prime 2025. The sequences generated in the laboratory (Pansloth149 and D50) were aligned with the sequences used in the 2020 study by Tung et al. [17].

### 2.7. Phylogenetic Analysis

A phylogenetic analysis was conducted using the 10-segment nucleotide sequences of the Pansloth 149 (p149) isolate detected in this study, as well as 20 available CGLV sequences from GenBank. The sequences of each segment were collated and aligned using MAFFT v7.490 [38]. After aligning each dataset, we used the maximum likelihood (ML) method in IQ-TREE v2.2.0 [39] to infer the phylogenetic relationships of each segment. Prior to running the analysis, we selected the most appropriate nucleotide substitution model for each dataset. The jModelTest plugin integrated into IQ-TREE was used to identify the best model based on the Bayesian Information Criterion (BIC) statistic. We then assessed the robustness of our phylogenetic tree with 1000 ultrafast bootstrap replicates [40]. The resulting phylogenetic trees were visualized and prepared for publication using FigTree version 1.4.4 (Andrew Rambaut, 2007, available at http://tree.bio.ed.ac.uk/, accessed on 15 May 2024). Amino acid-level changes were analyzed using MAFFT software (v. 1.5.0) for sequence alignment, followed by further analysis with Geneious Prime (v. 2025.0.2, Graphpad software LLC, Auckland, New Zealand) [38]. Finally, the visualization of amino acid changes was carried out using Snipit software (version 1.4).

## 3. Results

### 3.1. Characteristics of Sloths Collected

A total of 60 sloths, consisting of 91.7% *C. hoffmani* (*n* = 55) and 8.3% of *B. variegatus* (*n* = 5), were collected from 2013 to 2018 (Table 3). Most of the animals were captured in 2013 (38.3%) and in the Capira district, mainly in El Lidice, followed by Trinidad de las Minas village. Sloths were found in trees within village boundaries, approximately 500 m from residential areas and adjacent to agricultural land. These communities are located within patches of forest surrounded by agricultural fields producing corn, rice, beans, cassava, and plantains, and other typical regional agricultural products (Figure 1). The relatively small population also raises domestic animals for consumption, such as cattle, pigs, and poultry.

### 3.2. Plaque-Reduction Neutralization Test (PRNT)

From the tested sloth serum samples, 6.7% (95%, CI: 1.7–13.3%; *n* = 4/60) had neutralizing activity against the alphaviruses VEEV or MADV with low titers ranging from 1:20 to 1:80 (Table 4). None of the sloths had sera with neutralizing activity against both VEEV and MADV. All were negative for other alphaviruses (MAYV, CHIKV, and UNAV), suggesting that these antibodies were specific to each virus (Table S1). Of the sloths tested for Orthobunyavirus, 4.8% (95%, CI: 1.6–10.8%; *n* = 2/42) were seropositive for OROV, exhibiting very low titers of 1:20. Pansloth149 and D50 orbiviruses showed high seroprevalence in 23.3% (95%, CI:13.4–36.0%; *n* = 14/60) and 53.3% (95%, CI: 40.0–66.0%; *n* = 32/60) of the sloths, respectively, with high neutralizing titers ranging from 1:320 to 1:2560 (Table 4). Only six sloths had neutralizing activity against both orbiviruses (Table S1). None of the sloths had neutralizing antibodies against the flaviviruses DENV-2 and ZIKV. Similarly, antibodies against the phlebovirus PTV were not detected in these sera.

No significant associations were observed among the analyzed variables (sex, collection site, and species) (Table S2). Seropositive sloths were detected across all three study communities. VEEV-seropositive animals were found in Lidice (*n* = 2), Trinidad de las Minas (*n* = 1), and Las Pavas (*n* = 1). MADV-seropositive sloths were detected in Lidice (*n* = 2) and Trinidad de las Minas (*n* = 2). OROV-seropositive animals (*n* = 2) were found only in Lidice. For orbiviruses, D50-seropositive sloths were distributed across Lidice (*n* = 15), Trinidad de las Minas (*n* = 12), and Las Pavas (*n* = 5), while Pansloth149-positive animals were found in Lidice (*n* = 6), Trinidad de las Minas (*n* = 5), and Las Pavas (*n* = 3). Despite differences in urbanization levels and proximity to Panama City, no significant geographic clustering of specific virus seropositivity was observed (Table S2). This suggests that these arboviruses circulate widely across both peri-urban communities (Lidice, 60 km from Panama City) and more rural communities (Las Pavas and Trinidad de las Minas, 85–99 km from Panama City). Furthermore, viremia was not detected for any of the tested arboviruses by RT-PCR or viral isolation.

### 3.3. Pansloth 149 Is a Changuinola-like Virus

The molecular analysis of the Pansloth 149 (p149) viral isolate, including contig assembly and a comparison against public databases (Genbank sequences used in Tung et al. [17]), revealed ten RNA segments (GenBank accession numbers PV948253-PV948262). These segments displayed nucleotide and amino acid sequence identities with CGLV.

**Figure 2:**
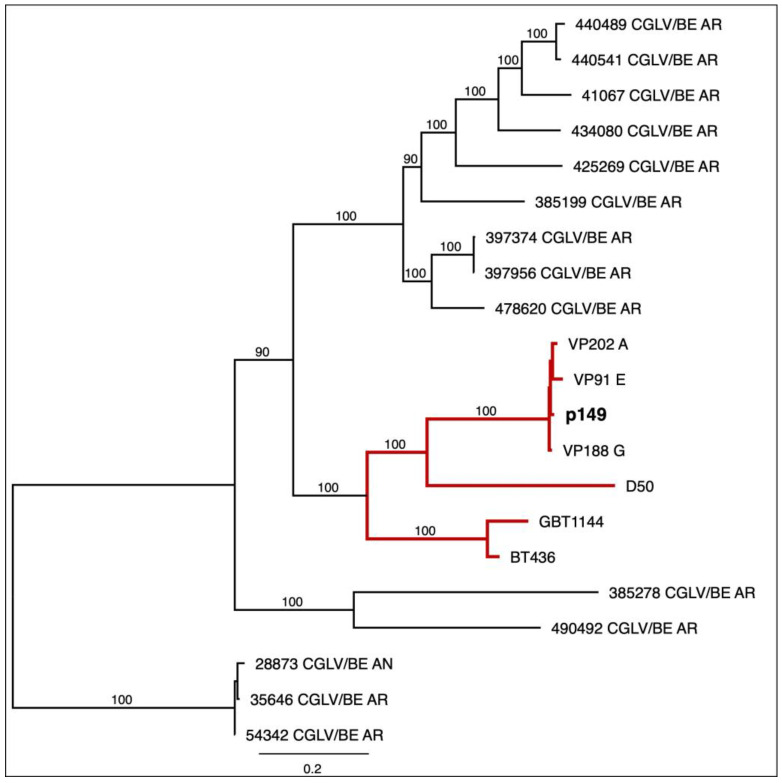
A phylogenetic tree was generated using the structural protein VP1 of CGLV strains. The tree was constructed with the IQ-TREE software version 2.2.2.6, using the GTR+F+I+R3 nucleotide substitution model determined by the software and with 1000 bootstraps. Only bootstrap support values greater than 90% are shown in the tree. Panamanian CGLV strains are indicated by red branches, and Pansloth 149 (p149) appears in bold letters. GenBank accession number: PV948253 (p149_S1). The red color shows the branches with Panamanian CGLV strains, including Pansloth 149 (p149).

Phylogenetic trees were constructed for various viral genome segments of orbiviruses D50 and Pansloth 149 (p149), including structural segments (VP1 (Figure 2), VP2 to VP7 (Supplementary Figure S1A–F)) and non-structural segments (NS1 to NS3) (Supplementary Figure S1G–I). Most segment-specific phylogenetic analyses showed that the p149 segments clustered with the Panamanian CGLV strains (BT436, GBT1144, D50, VP91 E, VP188 G, and VP202 A) (Figure 2, Supplementary Figure S1). Among the Panamanian strains, VP91 E, VP188 G, VP202 A, and p149 demonstrated the highest sequence identity, varying by approximately 3% across different segments. However, the VP2 segment from Pansloth 149 was closely related to D50, suggesting a possible reassortment (Supplementary Figure S1A).

Phylogenetic analysis revealed additional reassortment events beyond VP2 in Pansloth 149. The VP5 and NS1 segments displayed discordant phylogenetic patterns compared to most of the genome segments. The VP3, VP4, VP6, VP7, NS2, and NS3 segments of Pansloth 149 consistently clustered with VP91_E, VP188_G, and VP202_A (Supplementary Figure S1B,C,E,F,H,I), forming a cohesive Panamanian lineage with bootstrap support greater than 90%. However, the VP5 and NS1 segments showed distinct clustering patterns (Supplementary Figure S1D,G), with VP5 having a higher genetic distance to VP91E and VP188_G, whereas NS1 clustered with VP2020_A, close to BT436, GBT1144. These phylogenetic differences across multiple genome segments indicate that Pansloth 149 arose through several independent reassortment events rather than a single genome segment exchange.

**Figure 3:**
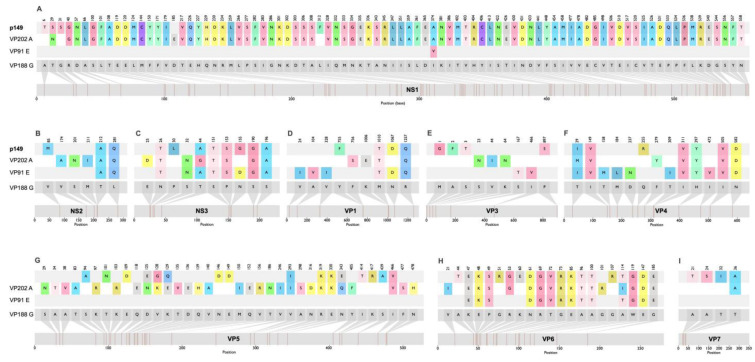
Amino acid comparison of PanSloth149 proteins with VP188 G strain as the reference. (**A**) NS1. (**B**) NS2. (**C**) NS3. (**D**) VP1. (**E**) VP3. (**F**) VP4. (**G**) VP5. (**H**) VP6. (**I**) VP7.

These four representative sequences—VP91 E, VP188 G, VP202 A, and Pansloth 149—were selected for a comparative amino acid analysis. The VP188 G protein sequence was used as the reference strain. A comprehensive table was generated to document the mutations in the viral proteins (Figure 3). A detailed amino acid comparison across all viral proteins revealed distinct variability patterns (Figure 3, Supplementary Figure S2). VP1, VP3, VP4, VP7, NS2, and NS3 showed limited mutations (fewer than 20 amino acid (a.a.) substitutions), while VP2, VP5, VP6, and NS1 exhibited substantially higher variability (more than 20 a. a. changes). The VP5, VP6, and NS1 proteins from Pansloth 149 showed the highest similarity to VP91_E proteins, followed by VP202_A; however, they displayed complete divergence from the reference strain VP188G (Figure 3). VP2 demonstrated the most extensive polymorphism, with mutations distributed across the entire length of the protein sequence (Supplementary Figure S2), affecting potential receptor-binding domains, antigenic sites, and structural features critical for viral entry [41].

## 4. Discussion

Our findings reveal that sloths (*C. hoffmanni* and *B. variegatus*) in rural and peri-urban areas of western Panama province are exposed to multiple arboviruses capable of causing human disease and epizootic epidemics. While none of the sloths tested were seropositive for antibodies against the major arboviruses responsible for urban outbreaks (DENV, CHIKV, and ZIKV), some were positive for endemic enzootic arboviruses (VEEV, MADV, and OROV). The circulation of these infections in wildlife near human settlements increases the probability of spillover, so our findings offer a key foundation to a One Health approach to surveillance and disease control where wildlife and human populations intersect.

The four sloths that tested positive for VEEV antibodies were different from those that tested positive for MADV, which rules out cross-reaction between these alphaviruses. Previous studies on VEEV seroprevalence in sloths from Central America reported results similar to our observation of 6.7% of VEEV seropositivity in western Panama. Sloths belonging to the same genera (*Bradypus* spp. and *Choloepus* spp.) with VEEV antibodies were found in Panamanian tropical forests during the 1980s [11,42]. In Costa Rica, VEEV seroprevalence reached 11% in these species in similar rural areas to those in our study [43]. VEEV and MADV maintain an enzootic life cycle, causing outbreaks in accidental hosts, such as humans and horses, mainly in eastern Panama (Darien province) [27,44,45,46]. Neutralizing antibodies against VEEV were detected in rodents and other mammals collected between 2011 and 2012, with a higher seroprevalence (27.3%) in El Cacao, in the Capira district of western Panama province, close to El Lídice and Trinidad de Las Minas [47], where we collected the sloths. The seroprevalence reported in rodents is higher than what we detected in sloths, suggesting that sloths may interact less with the VEEV transmission cycle than rodents, which are probably its main reservoir [48,49]. In this previous study in western Panama [47], no evidence of MADV viremia or antibodies was found in rodents or other mammals. Further research is needed to determine whether this is due to sloths having a higher interaction with the MADV cycle than other mammals or to a recent increase in its enzootic transmission cycle in this region. Interestingly, in Brazil, 4.5% of sloths captured between 2006 and 2008 in open areas bordering agricultural and forest areas tested positive for EEEV (the South American strain of EEEV, now reported as MADV) with antibody titers similar to those in our study (1:20) [50]. This MADV seroprevalence is similar to our report (6.7%), suggesting that sloths could interact with the MADV enzootic transmission cycle in different parts of the Americas. Future studies should compare VEEV and MADV seroprevalence in sloths with that of other sylvatic animals from the same communities in the western Panama province and the eastern Panama region. This will help determine if antibodies specific to these alphaviruses are associated with specific regions, land-use, or animal species. It will also help learn if there are changes in seroprevalence over time, which could suggest a change in viral circulation.

Despite their potential as reservoir hosts due to habitat overlap with known OROV vectors [51], few studies have examined serological evidence for OROV antibodies in sloths. Of the 42 sloths tested for OROV serology, only 2 had neutralizing antibodies with very low titers. Previous studies in Brazil and Panama (1960s–1970s) revealed the presence of neutralizing antibodies against OROV in two-toed sloths (*Choloepus hoffmanni*), suggesting prior exposure [42,52]. However, as in our study, neutralization titers were generally low, and sample sizes were small, making definitive conclusions about the role of sloths in the virus’s natural cycle difficult [42,52]. Although neutralization tests are the gold standard for identifying specific OROV antibodies, additional confirmatory testing is necessary to rule out cross-reactivity with related orthobunyaviruses [53]. It is also important to note that our assays were based on an older OROV strain, which is likely to be similar to the endemic Panamanian strains that circulated before the reassortant OROV strain causing the 2023–2025 American outbreak [54]. However, the temporal mismatch between the tested OROV strain and the circulating strain could affect antibody recognition. There is a notable knowledge gap regarding the role of sloths in OROV maintenance and transmission cycles, requiring studies focused exclusively on neutralization [55].

Antibodies against the arthralgic alphaviruses MAYV, CHIKV, and UNAV were not detected in the analyzed sloths. This result mirrors a previous Brazilian study in which sloths of other species (*B. torquatus* and *B. variegatus*) tested negative for MAYV and CHIKV antibodies, and no UNAV seroprevalence information was available [50].

Despite a recent outbreak during the samples’ collection years [56], no sloths were seropositive for the tested flaviviruses, including endemic DENV and emergent ZIKV. Unlike Brazilian studies that detected DENV antibodies in the sloth species *B. torquatus* and *B. variegatus* with a seroprevalence of 22.7% (5/22) for DENV-2, 9.1% for DENV-1, 22.7% for DENV-3, and 9.1% for DENV-4 [50], our study found no neutralizing antibodies against DENV-2. DENV-2 was the most prevalent serotype nationally during the collection period [13]. Future studies of DENV seroprevalence in sloths and other wild and domestic mammals living near humans are needed to understand viral exposure patterns. DENV has been detected in wild mosquitoes in some areas of the Americas [57], though the epidemiological significance of these findings remains to be determined. Currently, there is no evidence of a sylvatic DENV cycle in the Americas [58,59]. Furthermore, seroprevalence does not necessarily indicate that these species serve as viral reservoirs or contribute to transmission cycles, as is the case with West Nile virus seropositivity in humans and horses [60]. This distinction is particularly relevant for DENV, as it is primarily maintained through urban transmission cycles and does not require animal reservoirs for persistence [56].

Similarly, we did not detect neutralizing antibodies against the phlebovirus PTV, another endemic Panamanian arbovirus previously associated with sloths. This finding differs from a 1982 study conducted in the Majé, Cerro Azul, El Llano-Cartí, and Aguacate communities in the Panama province and eastern Panama region. In that study, PTV antibodies were found in 7.5% of *B. variegatus* (8/106) and in 16.7% of *C. hoffmanni* (11/66) [42,43]. A Costa Rican study also detected PTV antibodies in 32.4% of *C. hoffmanni* evaluated in 2006 [42]. PTV is typically transmitted by *Lutzomyia* spp. [43,61] and rarely infects humans, causing febrile illness [33]. In Colombia, a virus related to sloth phleboviruses was recently identified [62]. This finding suggests a probable relationship between sloths, *Lutzomyia*, PTV, and other phleboviruses. It also indicates that sloths may serve as reservoir hosts in the sylvatic transmission cycle of this sloth phlebovirus. Future longitudinal studies at the national and regional levels are needed to determine if PTV seroprevalence differs among sloth populations depending on the region and vector presence, and if it varies over time.

The orbiviruses Pansloth149 and D50, isolated from sloths at the GMI in the 1970s–1980s [17,18], showed seroprevalence of 25.0% and 75.0% in *Bradypus variegatus* and 31.0% and 69.0% in *Choleopus hoffmani*, respectively, with titers up to 1:2560. However, only 10% of sloths exhibited neutralizing activity against both orbiviruses. Future studies should determine the extent of cross-reactivity between them, as Pansloth149 and D50 are part of the Panamanian CGLV strain cluster. The high seroprevalence observed in this study, coupled with their isolation in sloths 50 years earlier, suggests that these orbiviruses circulate continuously among sloths, which could be their natural reservoir. No sloths exhibited apparent symptoms, and none had detectable virus. Further research is necessary to determine if these orbiviruses are pathogenic to sloths, if they can replicate in these animals at a sufficient level for vector transmission, and if they can infect and cause disease in humans.

In order to understand the interaction between sloths and these two orbiviruses, it is first necessary to characterize them genetically. Our sequencing analysis revealed that Pansloth149 (p149) segments belong to this Changuinola-like virus (CGLV) group of the *Sedoreoviridae* family and *Orbivirus* genus, which is characterized by high genetic variability. The viral strains BT436, GBT1144, D50, VP91 E, VP188 G, and VP202 A belong to a previously sequenced Panamanian strain group [17,18]. These strains are closely related genetically, suggesting common origin, recent common ancestor descent, or transmission within the same Panamanian population [18]. The VP1-based phylogenetic tree showed that the Pansloth 149 CGLV isolate shared a monophyletic root and was most closely related to the prototype strains VP188 G, VP 202 A, VP91 E, D50, and GBT1144, described in Panama by Tung et al. [17]. Our phylogenetic analysis provides compelling evidence that genomic reassortment plays a significant role in CGLV evolution in Panama. The Segment 2 of PanSloth 149, which codes for the VP2 protein, exhibited a distinct clustering pattern, clustering with D50 rather than with VP91 E, VP202 A, and VP188 G. Beyond the VP2 segment, the VP5 and NS1 segments in p149 exhibit phylogenetic divergence, indicating that this strain emerged through independent reassortment events rather than a single exchange. While VP1, VP3, VP4, VP6, VP7, NS2, and NS3 consistently cluster with VP91_E, VP188_G, and VP202_A with high bootstrap support (>90%), VP2, VP5 and NS1 show different phylogenetic patterns. These divergences across at least three genome segments suggest that co-infection with multiple CGLV strains occurs regularly in sloth populations and provides opportunities for segment exchange during viral replication. This interpretation is consistent with the high seroprevalence we observed for both D50 (53.3%) and Pansloth149 (23.3%), with 10% of sloths showing neutralizing antibodies against both viruses. This suggests concurrent or sequential exposure to multiple CGLV strains. Reassortment in segmented RNA viruses is a well-documented mechanism that generates genetic diversity and can alter host range, virulence, or transmission efficiency. This has been observed in other orbiviruses such as Bluetongue virus [63] and African horse sickness virus [64], where reassortment events have been linked to epidemic emergence and changes in pathogenicity.

Phan et al. previously identified reassortment among Panamanian CGLV strains [17], but the extent and functional implications remain poorly characterized. The VP2 segment, which encodes the cell attachment protein, exhibits the highest variability and is critical for host receptor binding and immune recognition [65]. Reassortment of the VP2 segment from a D50-like lineage into the Pansloth 149 backbone may represent an adaptive strategy for immune evasion or enhanced transmission efficiency in the sloth-sandfly transmission cycle. VP2 mediates virus-host attachment and antigen specificity and undergoes strong selective pressure driven by immune evasion and host-vector dynamics, particularly in ecological niches involving sloths and phlebotomine sandflies [65]. Future studies should determine whether segment 2 reassorted in PanSloth 149, when this occurred, and whether this resulted from selective pressure. Additionally, they should investigate whether reassortment of VP2 alters its recognition by neutralizing antibodies and determine the cross-reactivity of antibodies against PanSloth 149, D50, and the other Panamanian CGLV strains. Similarly, VP5 (which encodes an outer capsid protein) and NS1 (a nonstructural protein involved in viral replication and assembly) may contribute to fitness advantages through optimized virion assembly or replication kinetics. Understanding reassortment dynamics in CGLV and other orbiviruses is important for assessing spillover risk in urbanizing areas, because reassortment can generate novel variants with unpredictable phenotypic properties.

Comparative amino acid studies showed that the PanSloth 149 segments that code for the structural proteins (VP) VP1, VP3, VP4, VP6, VP7, and the nonstructural proteins (NS) NS2 and NS3 had an intermediate to high level of mutations when compared to their closest relatives, the VP202-A, V91_E, and VP188_G viruses. Previous studies on Bluetongue virus (BTV), a well-characterized *Orbivirus* of veterinary importance, demonstrated that the RGD (arginine–glycine–aspartate) tripeptide of VP7 is responsible for core attachment to *Culicoides* cells, highlighting its essential role in viral entry and cell tropism [66]. The amino acid mutations identified in VP7 from Pansloth 149 compared to other Panamanian CGLV are located at the N-terminal region of the protein (positions 21, 24, 32, and 36), notably far from the critical RGD tripeptide, which is conserved. Future studies should analyze how the different mutations of each protein affect viral transmission, the replication cycle, and pathogenesis. Identifying mutations through all the proteins, as well as reassortment of VP2, VP5, and NS1, may have significant functional implications.

The high variability of amino acids observed in VP2 may also have important functional implications. VP2 is the primary structural protein responsible for virus–host cell interactions, mediating receptor binding and cell penetration [67,68] and is therefore the principal target of neutralizing antibodies [69,70]. Our comparative amino acid analysis revealed extensive polymorphisms across the entire VP2 sequence of PanSloth 149, with over 200 amino acid substitutions compared to the VP188_G reference strain. This high level of polymorphism is similar to that described for VP2 of the Bluetongue virus [63,68,71]. These mutations, particularly in surface-exposed regions of VP2, could significantly impact antigenic properties and enable escape from neutralizing antibodies, which could have an impact on pathogenicity and infectivity in different hosts, as described for the *Orbivirus* prototype, the Bluetongue virus [72]. The diversity in VP2, which could translate into differences in susceptibility to neutralizing antibodies, could explain the differential neutralization patterns observed in our serological assays [72]. In these assays, only 10% of the sloths showed neutralizing activity against both Pansloth149 and D50, despite the fact that the VP2 coding segments of these orbiviruses are highly similar among Panamanian CGLV strains. These results suggest that mutations, as well as reassortment, might play an important role in CGLV diversity and the specificity of neutralizing antibodies against different orbiviruses.

The structural divergence in VP2, combined with its phylogenetic clustering with D50 rather than with VP91 E, VP202 A, or VP188 G, suggests that reassortment events have selected for VP2 variants with altered receptor-binding specificities or immune-escape capabilities [73,74]. The concentration of substitutions in domains likely involved in receptor recognition indicates positive selection pressure driven by host immune responses or vector adaptation [41]. The high variability of VP2, in contrast to the more conserved nature of VP1, VP3, VP4, and VP7, highlights the significance of this outer capsid protein in viral evolution. Further research is needed, including structural modeling and functional assays, to determine how these mutations on CGLV segments affect cell tropism, replication efficiency, antibody neutralization sensitivity, and potential advantages in evading host immunity or enhancing sandfly vector transmission [18,65,75].

In Panama, urbanization and deforestation increase the risk of infections associated with sloths as potential hosts for zoonotic pathogens and arboviruses [20]. The Lídice and Trinidad de las Minas communities, which had the highest sloth collection rates, are located in a heterogeneous landscape of secondary mixed broadleaf forests interspersed with agricultural areas. Forest fragmentation facilitates contact between sylvatic reservoirs and the human population [21] because it creates ecological edges where arthropod vectors maintain both sylvatic and peri-domestic transmission cycles, with high densities, facilitating arbovirus spillover [76,77,78].

Developing efficient monitoring and control programs requires understanding the role of sloths and other wild reservoirs in transmitting arboviral infections. In order to reduce the risk to public health while protecting biodiversity, strategies for monitoring, surveillance, and response must be strengthened using a One Health approach that integrates the community.

## Figures and Tables

**Table 1:** Geographic and demographic characteristics of the communities in Western Panama province.

Community	Subdistrict	District	Population Density (inhab./km^2^)	Distance from Panama City (km)	Coordinates	Altitude (m.a.s.I.)
Lidice	Lidice	Capira	149.5	60	8°45′ N, 79°54′ W	970
Trinidad de las minas	Cacao	Capira	31.2	85	8°46′32″ N, 79°59′45″ W	230
Las Pavas	Amador	La Chorrera	25	99	9°6′15″ N, 79°53′9″ W	50–156

Abbr.: km = kilometers; inhab. = inhabitants; m.a.s.I. = meters above sea level.

**Table 2:** Viral strains used in plaque-reduction neutralization test (PRNTs).

Viral Genus	Virus Species	Strain ID	Origin	Reference
Alphavirus	CHIKV	GMI-256137	GMI	[23]
	VEEV	TC-83	GMI/WRCEVA	[24]
	MADV	GMI-267113	GMI	[25]
	UNAV	BT-1495-3	GMI/WRCEVA	[26]
	MAYV	CH1	WRCEVA	[27]
Orthoflavivirus	DENV-2	429557 (NR-12216)	WRCEVA	[28]
	ZIKV	GMI-259249	GMI	[12]
Phlebovirus	PTV	GMI-483391	GMI	[29]
Orthobunyavirus	OROV	TVP-14297	WRCEVA	[30]
Orbivirus	D50	-	GMI/WRCEVA	[17]
	Pansloth149	GMI-p149	GMI/WRCEVA	—

Abbreviations: GMI, Gorgas Memorial Institute; WRCEVA, World Reference Center for Emerging Viruses and Arboviruses; CHIKV, chikungunya virus; VEEV, Venezuelan equine encephalitis virus; MADV, Madariaga virus; UNAV, Una virus; MAYV, Mayaro virus; DENV-2, dengue virus serotype 2; ZIKV, Zika virus; PTV, Punta Toro virus; OROV, Oropouche virus.

**Table 3:** Characteristics of sloths collected in the period from 2013 to 2018 (*n* = 60).

Characteristics	*N* (%)
**Year of collection**	
2013	23 (38.3%)
2014	13 (21.7%)
2015	6 (10.0%)
2018	18 (30.0%)
**Collection site**	
Lidice	27 (45.0%)
Las Pavas	10 (16.7%)
Trinidad de Las Minas	23 (37.3%)
**Species**	
*Choloepus hoffmanni*	55 (91.7%)
*Bradypus variegatus*	5 (8.3%)
**Sex**	
Female	34 (56.7%)
Male	26 (43.3%)

**Table 4:** Number of sloths positive samples with antibodies according to the titers by PRNT, 2013–2018, from western Panama.

Virus	Positive	Titers (PRNT)							
		1:20	1:40	1:80	1:160	1:320	1:640	1:1280	1:2560
VEEV (TC-83) *	4/60	-	3	1	-	-	-	-	-
b. MADV	4/60	4	-	-	-	-	-	-	-
c. OROV	2/42	2	-	-	-	-	-	-	-
d. Pansloth 149	14/60	1	7	-	2	3	1	-	-
e. D50	32/60	4	9	2	5	7	4	-	1

* A live-attenuated investigational vaccine for VEEV.

## Data Availability

The nucleotide sequence data supporting the results of this study have been deposited in GenBank under the following accession numbers: PV948253 (p149_S1), PV948254 (p149_S2), PV948255 (p149_S3), PV948256 (p149_S4), PV948257 (p149_S5), PV948258 (p149_S6), PV948259 (p149_S7), PV948260 (p149_S8), PV948261 (p149_S9), and PV948262 (p149_S10). These sequences are scheduled for public release on 1 August 2026, or upon publication, whichever occurs first. The data can be accessed through the National Center for Biotechnology Information (NCBI) GenBank database at https://www.ncbi.nlm.nih.gov/genbank/, accessed on 4 September 2025.

## Supplementary Materials

The following supporting information can be downloaded at: https://www.mdpi.com/article/10.3390/v17111507/s1, Table S1: PRNT_80_ neutralizing titers of sloths seropositive for each virus; Table S2: Characteristics associated with Arbovirus infection in sloths, bivariate analysis; Supplementary Figure S1: Phylogenetic tree of the CGLV nucleotide sequence, panels (A–I) represent segments 2 to 10, respectively; Supplementary Figure S2: Amino acid comparison of PanSloth149 VP2 protein with VP188 G strain as the reference.

## Abbreviations

The following abbreviations are used in this manuscript:

DENV	Dengue virus
ZIKV	Zika virus
VEEV	Venezuelan equine encephalitis
CGLV	Changuinola virus
PTV	Punta Toro virus
EEEV	Eastern equine encephalitis virus
MADV	Madariaga virus
OROV	Oropouche virus
CHIKV	Chikungunya virus
MAYV	Mayaro virus
GMI	Gorgas Memorial Institute
DIDETEC	Research and Technological Development Division
UTMB	University of Texas Medical Branch
WHO	World Health Organization
HI	Hemagglutination Inhibition
