# Supplementary material for: Seroprevalence of Arboviruses and Genetic Characterization of Orbiviruses in Sloths from Western Panama
Source: Viruses. 2025 Nov 17;17(11):1507. doi: 10.3390/v17111507 (PMC12656948; doi:10.3390/v17111507)
Supplement: Supplementary file 1 [file viruses-17-01507-s001.zip › viruses-3887673-supplementary.pdf]

**Supplementary Data.**

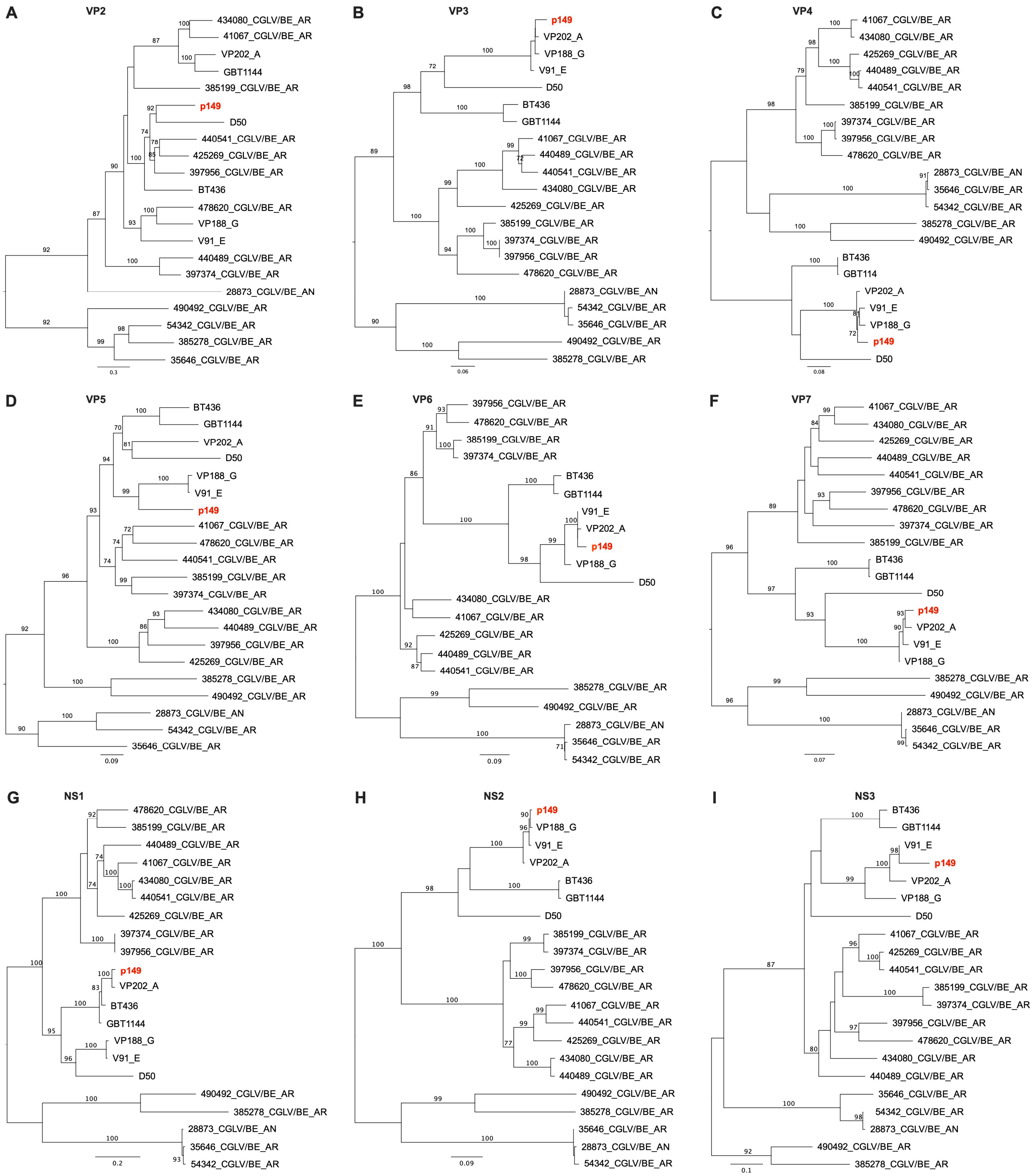

**Supplementary figure S1**. Phylogenetic tree of CGLV nucleotide sequence, panels (A-I) represent segments 2 to 10, respectively. The p149 strain is marked in red font. The phylogenetic tree was constructed by the maximum likelihood method using I!-TREE with substitution model selection (ModelFinder implemented in IQ-TREE) option and 1000 bootstraps. Bootstrap values are shown if >70%.

**Table S1. PRNT50 neutralizing titers of sloths seropositive for each virus**

| Sloth ID | VEEV (TC-83) | MADV | OROV | D50 | Pansloth149 |
|---|---|---|---|---|---|
| S-1 | - | - | 1:20 | 1:20 | - |
| S-2 | - | - | - | 1:40 | 1:40 |
| S-3 | - | - | - | 1:20 | - |
| S-5 | - | - | - | 1:40 | - |
| S-7 | - | - | - | 1:320 | - |
| S-8 | - | - | - | - | 1:40 |
| S-9 | - | - | - | 1:40 | - |
| S-12 | - | - | - | - | - |
| S-13 | - | - | - | 1:320 | 1:20 |
| S-14 | - | - | - | 1:160 | - |
| S-15 | - | - | - | 1:40 | - |
| S-16 | - | 1:20 | - | - | - |
| S-18 | 1:40 | - | - | - | - |
| S-20 | 1:40 | - | - | - | - |
| S-21 | - | - | - | - | 1:320 |
| S-22 | 1:40 | - | - | - | - |
| S-23 | - | - | - | 1:80 | - |
| S-24 | 1:80 | - | - | 1:160 | - |
| S-25 | - | - | - | 1:40 | - |
| S-27 | - | - | - | 1:40 | - |
| S-30 | - | - | - | 1:320 | - |
| S-31 | - | - | - | 1:320 | - |
| S-32 | - | - | 1:20 | 1:640 | - |
| S-34 | - | - | - | 1:160 | - |
| S-37 | - | 1:20 | - | 1:20 | 1:640 |
| S-39 | - | - | - | 1: 2 560 | - |
| S-41 | - | - | - | 1:320 | - |
| S-42 | - | - | - | 1:80 | - |
| S-43 | - | - | ND | - | 1:40 |
| S-44 | - | - | ND | 1:40 | 1:160 |
| S-45 | - | - | ND | 1:320 | - |
| S-46 | - | - | ND | 1:160 | 1:320 |
| S-47 | - | - | ND | 1:40 | 1:160 |
| S-48 | - | - | ND | 1:640 | 1:320 |
| S-49 | - | - | ND | - | 1:40 |
| S-50 | - | - | ND | 1:640 | 1:40 |
| S-52 | - | - | ND | 1:640 | - |
| S-53 | - | - | ND | 1:160 | 1:40 |

| | | | | | |
|---|---|---|---|---|---|
| S-54 | - | - | ND | 1:40 | - |
| S-55 | - | - | ND | - | 1:40 |
| S-56 | - | - | ND | 1:20 | - |
| S-57 | - | 1:20 | ND | - | - |
| S-58 | - | - | ND | - | - |
| S-59 | - | 1:20 | ND | 1:320 | - |
| S-60 | - | - | ND | - | - |

"-"=negative, "ND"=not done because of low sera volume.

**Table S2. Characteristics associated with Arbovirus infection in sloths, bivariate analysis*.**

| Characteristics | | Arbovirus seroprevalence | | p |
|---|---|---|---|---|
| | | Absence (n=15) **n (%)** | Presence (n=45) **n (%)** | |
| **Sex** | | | | 0.655 |
| | Male | 6 (7.5) | 24 (22.5) | |
| | Female | 8 (6.5) | 18 (19.5) | |
| | Unknown | 1 (1.0) | 3 (3.0) | |
| **Collect site** | | | | 0.265 |
| | Lidice | 4 (6.8) | 23 (20.2) | |
| | Las Pavas | 3 (2.5) | 7 (7.5) | |
| | Trinidad de las M. | 8 (5.8) | 15 (17.2) | |
| **Species** | | | | 0.591 |
| | Choloepus hoffmanni | 13 (13.8) | 42 (41.2) | |
| | Bradypus variegatus | 2 (1.2) | 3 (3.8) | |

***Bivariate analysis; Chi-square (Chi2).**

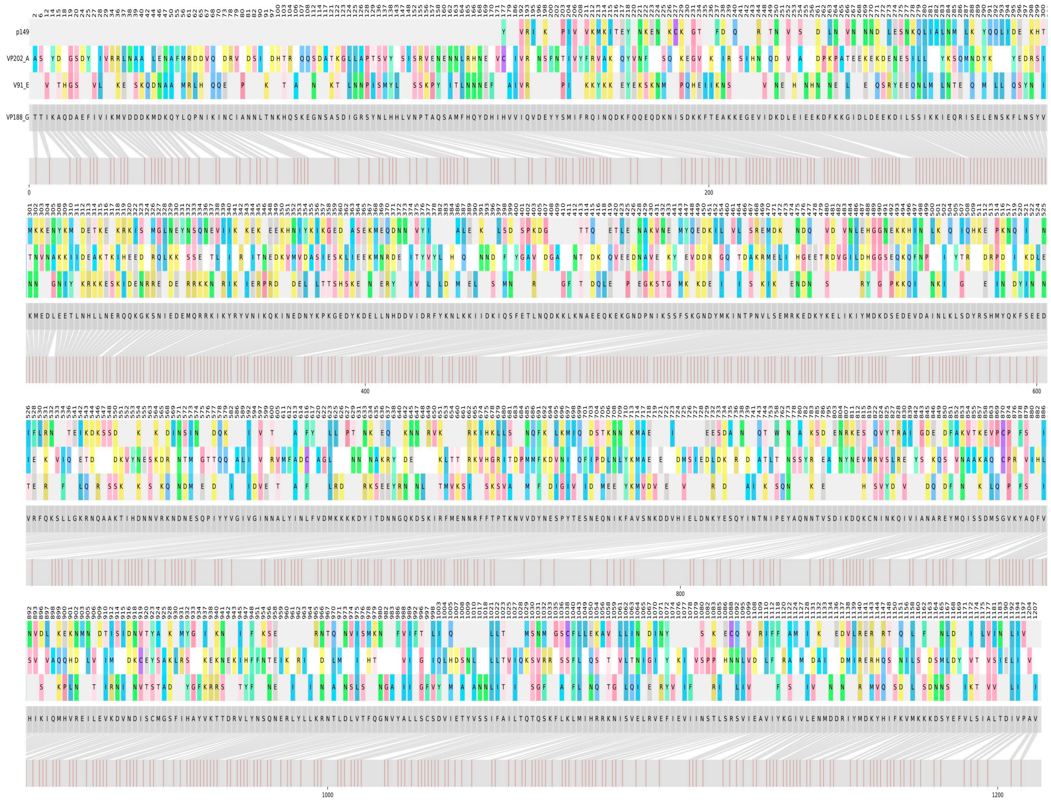

**Supplementary figure S2.** Amino Acid Comparison of PanSloth149 VP2 protein with VP188 G strain as the Reference.
